# Gray matter abnormalities and memory impairment in left mesial temporal lobe epilepsy

**DOI:** 10.3389/fnhum.2025.1554091

**Published:** 2025-08-14

**Authors:** Agnieszka Olejnik, Aleksandra Bala, Weronika Rejner, Antonina Gottman-Narożna, Andrzej Rysz, Maja Kopytek-Beuzen, Patrycja Naumczyk, Przemysław Kunert

**Affiliations:** ^1^Faculty of Psychology, University of Warsaw, Warsaw, Poland; ^2^Department of Neurosurgery, Medical University of Warsaw, Warsaw, Poland; ^3^Department of Neurosurgery, 1 Military Clinical Hospital with Outpatient Clinic: Lublin, Subsidiary in Ełk, Ełk, Poland; ^4^University of Gdańsk, Gdańsk, Poland

**Keywords:** mesial temporal lobe epilepsy (MTLE), left mesial hippocampal sclerosis, memory deficits, short-term memory, long-term memory, working memory, gray matter, volumetry

## Abstract

**Objectives:**

Mesial temporal lobe epilepsy (MTLE) is a common neurological disorder, with memory impairment being one of its typical symptoms. Most previous studies have focused on assessing declarative memory directly related to hippocampal functions, but emerging data suggest a decline in the efficiency of other types of memory as well. The aim of this study was to comprehensively assess various types of memory and analyze the relationship between memory performance and the volume of selected gray matter structures.

**Methods:**

In total, 21 patients with left-MTLE and 28 age- and education-matched healthy individuals underwent neuropsychological assessment using the Wechsler Memory Scale IV (WMS-IV) to evaluate memory functioning. Magnetic resonance imaging was also conducted to assess gray matter volume and structure in all participants.

**Results:**

Compared with healthy controls, patients with left-MTLE showed significantly reduced performance in short-term verbal and visual memory, long-term verbal and visual memory, and working memory. Volumetric analysis revealed differences in gray matter volume between groups, with some structures being smaller and others larger in the patient group. Numerous correlations were found between WMS-IV scores and the volume of specific brain regions. Significant associations were observed both ipsilateral and contralateral to the epileptic focus, involving regions such as the cerebellar cortex, cingulate gyrus, insula, thalamus, and pallidum.

**Conclusion:**

This study expands our understanding of the memory profile in patients with MTLE. Neuropsychological testing showed that patients performed worse than controls across all assessed memory domains. Additionally, the study identified a distinct pattern of neuronal abnormalities and brain–behavior correlations. These findings suggest that the extent of structural brain anomalies may be linked to the severity of memory impairment in MTLE, underscoring the complex relationship between neuroanatomy and cognitive function in this population.

## 1 Introduction

Mesial temporal lobe epilepsy (MTLE) is a common type of epilepsy, often caused by hippocampal sclerosis and frequently resistant to antiepileptic drugs (Ryvlin et al., 2014; Siemianowski and Królicki, 2005; Kwan and Sander, 2004). The mesial portion of the temporal lobe is strongly associated with memory, as evidenced by numerous studies documenting mnestic impairment in this patient population (Butler and Zeman, 2008; Rayner et al., 2016; Zhu et al., 2021). Although seizures originate in a specific focus, they may impact the structure and function of the entire brain because epilepsy is a network disease (Bonilha et al., 2012). This has been demonstrated by studies identifying neural damage extending beyond the hippocampus to regions such as the amygdala and parahippocampal gyrus (Dos Santos Silva et al., 2024; Zheng et al., 2018; Pail et al., 2010), as well as the corpus callosum, prefrontal cortex, thalamus, caudate nucleus, cingulate gyrus, and insula (Dos Santos Silva et al., 2024; Zheng et al., 2018; Bonilha et al., 2012). Therefore, structural changes in patients with MTLE are not limited to the mesial temporal lobe but can also be found throughout the brain, even in the hemisphere opposite the epileptic focus (Pail et al., 2010; Bonilha et al., 2012). As a result, patients experience a range of difficulties beyond memory impairment, including deficits in executive functions, attention, language, social cognition, and more (Olejnik et al., 2024; Zhou et al., 2019; Bell et al., 2003; Bala et al., 2024; Ives-Deliperi and Butler, 2021). Memory deficits, in particular, cannot be explained solely by changes in the hippocampus because memory is a highly complex construct. There is no single brain region responsible for all memory processes.

Memory is a complex and broad concept, encompassing many subtypes. However, in this study, we focused on specific types of memory: short-term verbal memory, short-term visual memory, long-term verbal memory, long-term visual memory, and working memory.

Epileptic seizures may be related to structural changes in the brain, with a potentially bidirectional relationship between the two variables. These structural alterations, in turn, may correlate with the clinical presentation and cognitive impairment profiles of patients. Several studies have shown that seizures can disrupt various types of memory (Butler and Zeman, 2008; Rayner et al., 2016; Zhu et al., 2021; Caciagli et al., 2023). Therefore, when assessing patient functioning, it is important to look beyond the most obvious and frequently studied domain—declarative long-term memory (Ono et al., 2021; Zheng et al., 2018; Butler and Zeman, 2008)—and expand the diagnostic protocol to include tasks that evaluate other types of memory. It is also essential to assess memory in at least two modalities: verbal and visual. Given the breadth and complexity of the memory construct and its various subtypes, this study was designed to allow for a comprehensive examination of memory. In light of potential structural abnormalities, we also conducted volumetric analyses of gray matter in patients’ brains, measuring the volume of individual regions. These measurements were then used for both comparative analyses (with a group of healthy individuals) and correlational analyses (with results from comprehensive memory assessments).

The aims of this study were to comprehensively evaluate various aspects of memory in patients with drug-resistant MTLE and compare them with healthy subjects, to correlate memory performance with the volume of specific brain structures, and to examine the relationship between memory performance and clinical factors such as age at epilepsy onset and duration of epilepsy.

## 2 Materials and methods

### 2.1 Participants

In total, 31 patients diagnosed with MTLE and 28 healthy controls participated in this study. Patients were recruited from the Department of Neurosurgery at the Medical University of Warsaw during hospitalization. The diagnosis of MTLE was confirmed by an experienced epileptologist based on seizure semiology, video-electroencephalography, and magnetic resonance imaging (MRI) findings that revealed hippocampal abnormalities suggestive of hippocampal sclerosis. The control group was recruited through a social media announcement. Healthy individuals were matched with the clinical group in terms of age and level of education. The exclusion criteria were the presence of metal elements in the body or claustrophobia preventing MRI examination; significant visual, auditory, or motor impairments that could interfere with neuropsychological testing; neurological diseases (other than epilepsy in the clinical group); use of medications affecting the central nervous system (excluding antiepileptic drugs in the clinical group); history of severe head injury; frequent alcohol consumption (several times per week); and use of other psychoactive substances. For the clinical group, additional exclusion criteria included having more than one epileptic focus and having undergone surgical treatment for epilepsy. Because of the small number of patients with right-hemisphere epileptic foci, which was insufficient for robust analysis, we chose to conduct analyses exclusively on patients with a left-sided focus (left-MTLE). Further details are provided in Table 1.

**TABLE 1:** Demographic and clinical characteristics of patients with left-MTLE and controls.

	Left-MTLE group	Control group
N	21	28
Age (years)	39.71 (10.2)	35.71 (10.7)
Years of education	14.38 (2.7)	14.21 (2.3)
Epilepsy duration (years)	21.42 (8.8)	n/a
Age at epilepsy onset	17.62 (13.3)	n/a
Frequency of focal seizures (monthly)	14.29 (24.2)	n/a
Frequency of generalized seizures (annually)	4.14 (9.8)	n/a

Data presented as mean (SD); n/a, not applicable.

### 2.2 Procedure

All participants were examined individually over two separate sessions. The first session involved providing general information about the study and obtaining written informed consent, followed by a brief interview to collect demographic information and a neuropsychological assessment focused on evaluating memory functions. Patients with MTLE were also asked to provide information about their seizures and medical history. This session lasted approximately 60–90 min, varying between participants depending on their condition. The examination took place in a quiet, isolated, and well-lit room at the hospital. The second session involved MRI scanning, which all participants underwent. This part lasted approximately 1 h. All procedures were conducted in accordance with the Declaration of Helsinki and were approved by the Ethics Committee of the Faculty of Psychology at the University of Warsaw. All participants provided informed consent and received financial compensation for their participation.

### 2.3 Neuropsychological examination

To thoroughly assess participants’ memory function, the Fourth Edition of the Wechsler Memory Scale (WMS-IV) was administered. The WMS-IV is a battery designed to evaluate various aspects of memory and working memory in adults. It assesses auditory memory, visual memory, visual working memory, immediate memory, and delayed memory. The WMS-IV includes seven subtests, four of which contain both immediate and delayed recall components (Wechsler, 2009).

#### 2.3.1 Visual reproduction I

This subtest assesses non-verbal visual memory. The examiner presents a series of five designs, shown one at a time in a booklet for 10 s each. After viewing each design, the examinee is asked to draw it from memory as accurately as possible. There is no time limit for the drawing task.

#### 2.3.2 Visual reproduction II

This subtest is administered 20–30 min after VR I and assesses long-term visuospatial memory. In the delayed condition, the examinee is asked to recall and draw from memory the designs they previously drew, in any order. After completing the free recall portion, the recognition task is administered. The examiner presents six different designs for each of the five original items, and the examinee must identify which design matches the one they were asked to remember in the VR I task.

#### 2.3.3 Logical memory I

This subtest assesses narrative memory under free recall conditions. The examiner orally presents two short stories, one at a time. Immediately after each presentation, the examinee is asked to repeat the story, including as many details as they can recall.

#### 2.3.4 Logical memory II

This subtest is administered 20–30 min after LM I and assesses long-term narrative memory through both free recall and recognition tasks. The examinee is asked to recall both stories presented in the first part (LM I). After completing the free recall, the recognition portion is administered, during which the examinee answers 15 yes/no questions about each story.

#### 2.3.5 Spatial addition

This subtest assesses visuospatial working memory through a visual addition task. The examinee is shown two 4 × 4-cell grids from the Stimulus Booklet, each containing blue and red dots, presented one after the other for 5 s each. The examinee is instructed to remember the placement of the blue dots on each grid and to ignore the red dots. After viewing both grids, the examinee’s task is to place blue and white dots on a Memory Grid according to a specific set of rules: blue dots should be placed in the same positions as those shown across both grids; white dots should be placed only where blue dots appeared in the same location on both grids. The examinee is also provided with red dots, which should not be placed on the grid. The test is discontinued after three consecutive incorrect responses.

#### 2.3.6 Verbal paired associates I

This subtest assesses verbal memory based on associative learning. The examiner reads 14 word pairs (e.g., sky–cloud), then reads the first word of each pair, and the examinee is asked to provide the corresponding word. Each response must be given within 5 s. There are four trials using the same list of word pairs, but presented in a different order each time. If the examinee gives an incorrect response, the correct answer is provided.

#### 2.3.7 Verbal paired associates II

This subtest is administered 20–30 min after VPA I and assesses long-term recall of verbally paired information. The examinee is presented with the first word of each of the 14 word pairs learned in VPA I and asked to recall the corresponding second word. In this condition, the word pairs are not read aloud by the examiner, and no cues are provided. The examinee has 10 s to respond to each item. Following the free recall portion, the recognition task is administered. In this task, the examinee is shown 40 word pairs and must identify whether each pair was part of the original list from the immediate condition, responding with yes or no.

#### 2.3.8 Designs I

This subtest assesses spatial memory for visual material. The examiner presents a 4 × 4-cell grid containing 4–8 designs (depending on the condition) for 10 s, then removes it from the examinee’s view. The examinee must then select the designs from a set of 8 to 16 cards (also depending on the condition) and place them on a Memory Grid in the same locations as originally shown. The task includes four conditions of increasing difficulty: the first with four designs to remember, the second and third with six designs, and the fourth with eight designs.

#### 2.3.9 Designs II

This subtest is administered 20–30 min after DE I and assesses long-term spatial and visual memory through free recall and recognition tasks. The examinee is asked to select cards from the immediate condition and place them on the Memory Grid in the same positions as before. Following the free recall task, the examiner presents a series of grids containing various designs and asks the examinee to identify the two designs that match both the shape and placement from the immediate condition (DE I).

#### 2.3.10 Symbol span

This subtest assesses visual working memory using novel visual stimuli. The examiner presents a series of abstract symbols on a page for 5 s. Then, a new page is shown with various symbols, and the examinee is asked to identify the symbols from the previous page and select them in the same order in which they were originally presented in the stimulus booklet. The number of symbols increases gradually throughout the task.

#### 2.3.11 Additional memory indicators

To assess memory retention, an additional indicator was calculated: the sum of points scored in each subtest examining long-term memory (LM II, VPA II, VR II, DE II) was divided by the sum of points obtained in the corresponding immediate recall subtests (LM I, VPA I, VR I, DE I) and multiplied by 100. This yielded a percentage representing the proportion of stored information.

### 2.4 Neuroimaging data acquisition

MRI scans were performed using a 3.0 Tesla Skyra (Siemens) MRI scanner with a 16-channel head coil at the Department of Radiology, Medical University of Warsaw. All participants were scanned after completing the neuropsychological assessment. A three-dimensional brain volume imaging (3D-BRAVO) sequence was used to acquire structural images with the following parameters: repetition time (TR) = 2,200 ms, echo time (TE) = 4.94 ms, inversion time (TI) = 1,100 ms, flip angle (FA) = 7°, matrix size = 256 × 256, voxel size = 1 × 1 × 1 mm^3^, and 176 slices. A three-dimensional fluid-attenuated inversion recovery sequence was also performed with the following parameters: TR = 5,000 ms, TE = 371 ms, FA = 120°, matrix size = 256 × 256, voxel size = 1 × 1 × 1 mm^3^, and 176 slices.

### 2.5 Anatomical data processing

Raw T1-weighted DICOM images were reviewed and converted to NIfTI format using MRIcron’s dcm2niix (Li et al., 2016). Standard volumetric and surface processing pipelines were then implemented in FreeSurfer (version 7.2.0), as described in detail in the authors’ original papers (see: Dale and Sereno, 1993; Dale et al., 1999; Fischl et al., 1999; Fischl and Dale, 2000). Briefly, the procedures included skull stripping, B1-bias field correction, and gray–white matter segmentation, followed by non-linear registration of individual images to stereotaxic atlases, which provided labels for brain regions. The standard FreeSurfer atlas was used for volumetric estimation of subcortical structures (Fischl et al., 2002), and the Desikan–Killiany anatomical atlas was used for cortical surface measurements (Desikan et al., 2006). Each patient’s data underwent additional visual inspection to confirm the accuracy of the anatomical labeling. All volumetric measures were adjusted for individual estimated intracranial volume to account for inter-subject differences in brain size.

### 2.6 Statistical analysis

Statistical analyses were conducted using IBM SPSS Statistics for Windows, version 29.0.2 (IBM Corp., Armonk, NY, United States). The Shapiro–Wilk test confirmed that the data followed a normal distribution, and other assumptions for parametric testing were also met. The independent-samples Student’s *t*-test was used to compare mean scores between patients and healthy controls. Pearson’s r coefficient was used to analyze correlations between continuous variables. To reduce the risk of false positives, a Bayesian local false discovery rate (FDR) correction was applied (threshold < 0.2), and significant results were obtained for each type of memory.

## 3 Results

### 3.1 Participant characteristics

Descriptive statistics for both groups are presented in Table 1. There were no significant differences in age (*t* = −1.320, *p* = 0.097) or years of education (*t* = −0.232, *p* = 0.409), allowing us to assume that any differences observed in neuropsychological test performance or gray matter volumetric properties were attributable to group effects rather than demographic differences.

### 3.2 Comparison of memory indicators in patients with MTLE and healthy controls

Intergroup comparisons revealed significant differences across most WMS indicators, with large or very large effect sizes in the majority of cases (Table 2).

**TABLE 2:** Between-group comparisons of WMS-IV indicators.

WMS-IV subtest	left-MTLE *N* = 21 M (SD)	HC *N* = 28 M (SD)	*t*	*p*	Cohen’s d
LM I	23.10 (7.80)	31.50 (7.32)	−3.87	< 0.001	−1.12
LM II	17.19 (7.96)	29.18 (7.28)	−5.48	< 0.001	−1.58
LM II recognition	23.48 (3.49)	26.21 (3.20)	−2.85	0.003	−0.82
% LM	71.91 (22.07)	93.83 (10.63)	−4.05	< 0.001	−1.28
VPA I	21.33 (8.45)	35.54 (10.86)	−4.97	< 0.001	−1.43
VPA II	6.14 (3.20)	10.38 (2.77)	−4.94	< 0.001	−1.43
VPA II recognition	35.52 (2.52)	38.86 (1.21)	−6.13	< 0.001	−1.77
% VPA	83.83 (20.91)	92.55 (13.32)	−1.78	0.041	−0.51
VR I	32.24 (6.24)	37.79 (3.77)	−3.61	< 0.001	−1.12
VR II	22.00 (7.94)	30.29 (8.47)	−3.48	< 0.001	−1.00
VR II recognition	5.33 (1.28)	6.14 (1.11)	−2.37	0.011	−0.68
% VR	67.68 (19.99)	79.53 (18.41)	−2.15	0.018	−0.62
DE I	60.19 (13.08)	76.96 (16.48)	−3.84	< 0.001	−1.11
DE II	50.29 (11.79)	63.96 (18.12)	−3.01	0.002	−0.87
DE II recognition	14.35 (2.11)	16.46 (3.23)	−2.74	0.004	−0.75
% DE	84.62 (17.29)	82.95 (12.67)	0.39	0.349	1.11
SA	11.24 (3.94)	15.75 (3.60)	−4.17	< 0.001	−1.21
SSP	15.76 (4.72)	25.00 (7.35)	−5.34	< 0.001	−1.45

Data presented as mean (SD). Statistically significant results are shown in bold. DE, designs; LM, logical memory; SD, standard deviation; SA, spatial addition; SSP, symbol span; VPA, verbal paired associates; VR, visual reproduction.

**FIGURE 1:**
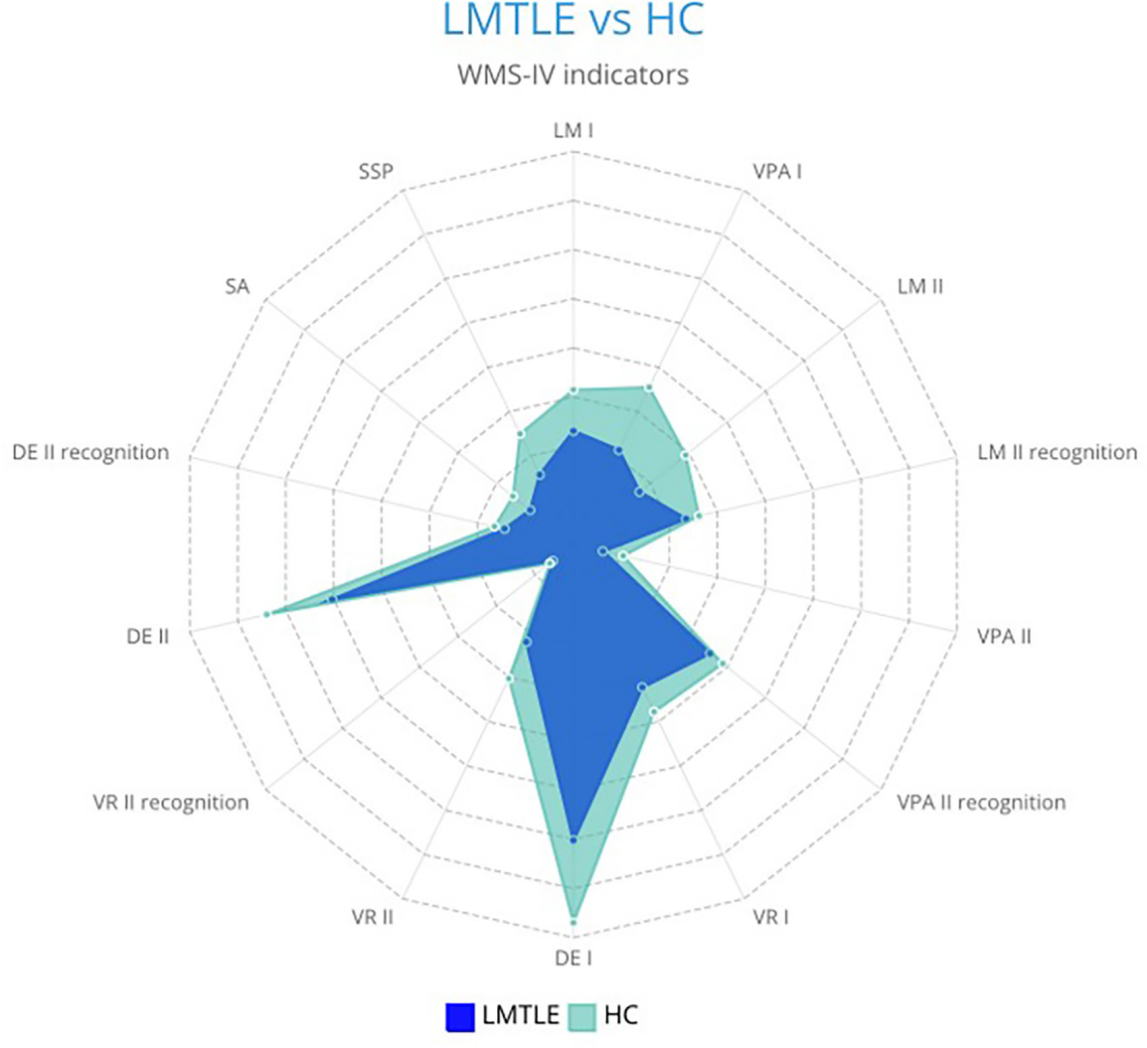
Differences in WMS-IV memory indicators between patients with left-MTLE (LMTLE) and healthy controls (HC).

The results obtained from the WMS-IV performance by the left-MTLE group and the control group are shown in Figure 1.

### 3.3 Comparison of structural gray matter measures in patients with left-MTLE and healthy controls

Statistically significant differences were found in intergroup comparisons between healthy controls and patients with left-MTLE in the following gray matter regions:

- Left hemisphere: cuneus (*t* = 2.397, *p* = 0.010), lateral occipital cortex (*t* = 3.233, *p* = 0.001), superior temporal gyrus (*t* = −1.810, *p* = 0.040), temporal pole (*t* = −2.397, *p* = 0.010), and hippocampus (*t* = −1.926, *p* = 0.032).
- Right hemisphere: caudal anterior cingulate gyrus (*t* = 1.890, *p* = 0.032), pars orbitalis (*t* = −2.038, *p* = 0.024), posterior cingulate gyrus (*t* = 2.071, *p* = 0.022), caudate nucleus (*t* = 1.726, p = 0.045), pallidum (*t* = −1.893, *p* = 0.032), and amygdala (*t* = 1.701, *p* = 0.048).

Bilateral differences were also observed in the cerebellar cortex: left cerebellum cortex (*t* = −1.763, *p* = 0.042) and right cerebellum cortex (*t* = −2.158, *p* = 0.018).

Regions that were significantly smaller in patients with left-MTLE compared with healthy subjects included the left superior temporal gyrus, left temporal pole, left hippocampus, right pars orbitalis, right pallidum, and bilateral cerebellum cortex. By contrast, regions that were significantly larger in patients with left-MTLE included the left cuneus, left lateral occipital cortex, right caudal anterior cingulate gyrus, right posterior cingulate gyrus, right caudate nucleus, and right amygdala.

### 3.4 Gray matter areas associated with memory performance in left-MTLE population

Correlation analyses were conducted within the left-MTLE group to explore the relationships between memory test performance and gray matter abnormalities. To enhance interpretability, WMS-IV subtests were grouped according to the specific type of memory they assessed:

- Short-term verbal memory: LM I and VPA I
- Short-term visual memory: VR I and DE I
- Long-term verbal memory: LM II, LM II recognition,% LM, VPA II, VPA II recognition, and% VPA
- Long-term visual memory: VR II, VR II recognition,% VR, DE II, DE II recognition, and% DE
- Working memory: SA and SSP

**FIGURE 2:**
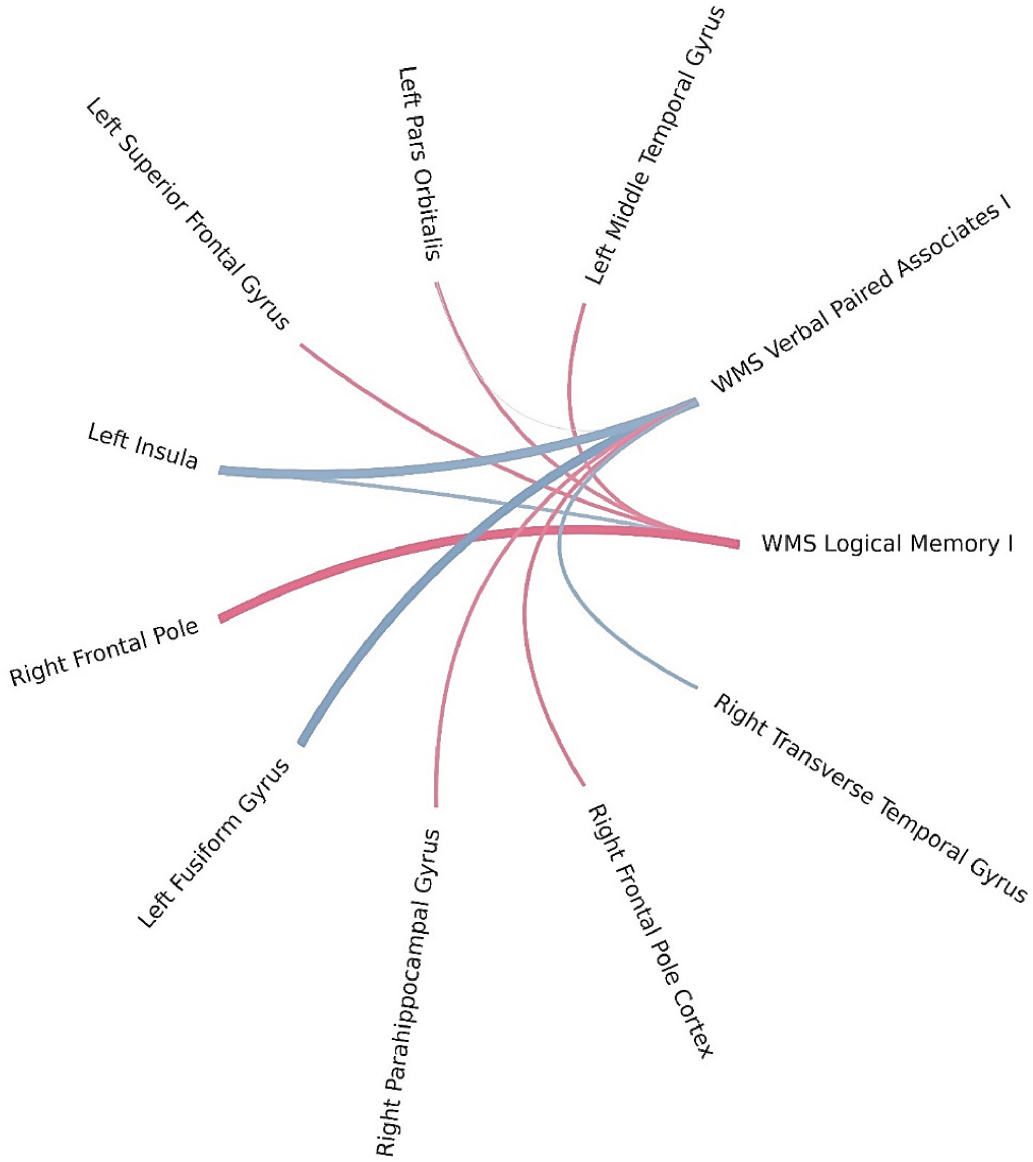
Brain regions correlated with WMS-IV short-term verbal memory indicators in the left-MTLE patient group. The blue lines represent positive correlations and the red lines represent negative correlations, the thickness of the lines reflects the strength of the correlation.

Using Bayesian local FDR correction (threshold < 0.2), significant correlations were identified between short-term verbal memory performance and cortical thickness (see Table 3; Figure 2). Specifically, in patients with left-MTLE, short-term verbal memory scores were associated with cortical thickness in the following regions: the fusiform gyrus, insula, middle temporal gyrus, pars orbitalis, and superior frontal gyrus in the left hemisphere, and the frontal pole, parahippocampal gyrus, and transverse temporal gyrus in the right hemisphere.

**TABLE 3:** Gray matter areas associated with short-term verbal memory.

Subtest	Brain region	*r*	*p*	Local FDR
WMS LM I	LH middle temporal gyrus	−0.468	0.032509	0.033591
	LH pars orbitalis	−0.457	0.037217	0.040917
	LH superior frontal gyrus	−0.472	0.030723	0.031697
	LH insula	0.448	0.04164	0.051126
	RH frontal pole	−0.552	0.009531	0.058838
WMS VPA I	LH pars orbitalis	0.0524	0.82159	0.139467
	LH fusiform gyrus	0.583	0.00554	0.004859
	LH insula	0.521	0.015473	0.042904
	RH parahippocampal gyrus	−0.454	0.038505	0.043539
	RH frontal pole cortex	−0.484	0.026212	0.029118
	RH transverse temporal gyrus	0.49	0.024089	0.029132

RH, right hemisphere; LH, left hemisphere; LM, logical memory; VPA, verbal paired associates.

**FIGURE 3:**
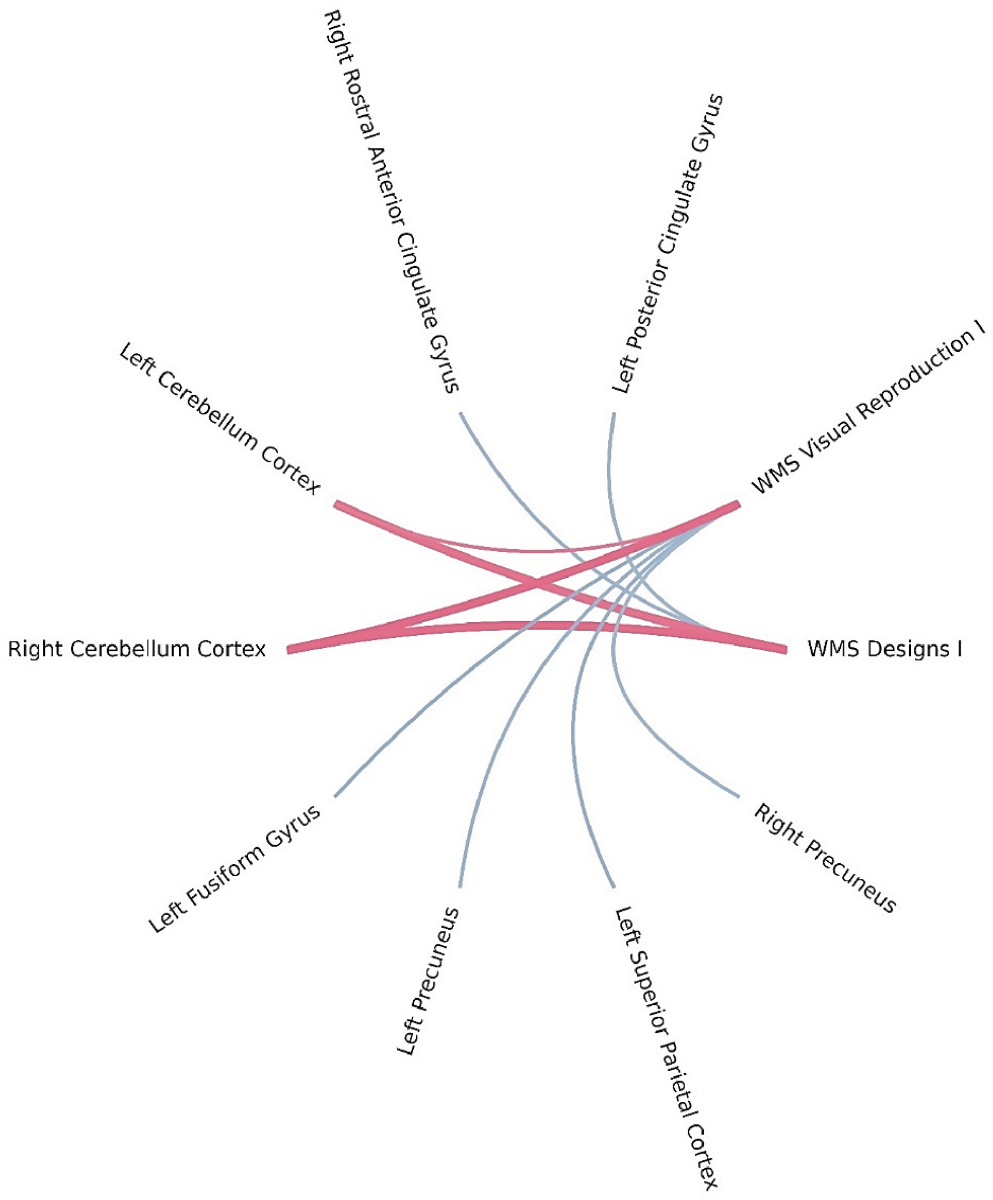
Brain regions correlated with WMS-IV short-term visual memory indicators in the left-MTLE patient group. The blue lines represent positive correlations and the red lines represent negative correlations, the thickness of the lines reflects the strength of the correlation.

Bayesian local FDR analysis (threshold < 0.2) revealed significant correlations between short-term visual memory scores and brain structural measures (Table 4; Figure 3). Short-term visual memory was associated with the following regions: fusiform gyrus, precuneus, superior parietal cortex, and posterior cingulate gyrus in the left hemisphere; precuneus and rostral anterior cingulate gyrus in the right hemisphere; and the cerebellar cortex bilaterally.

**TABLE 4:** Gray matter areas associated with short-term visual memory.

Subtest	Brain region	*r*	*p*	Local FDR
WMS DE I	LH posterior cingulate gyrus	0.443	0.044285	0.06088
	RH rostral anterior cingulate gyrus	0.455	0.038292	0.04592
	Left cerebellum cortex	−0.539	0.011617	0.078387
	Right cerebellum cortex	−0.56	0.008286	0.007663
WMS VR I	LH fusiform gyrus	0.486	0.025432	0.039662
	LH precuneus cortex	0.454	0.03881	0.04692
	LH superior parietal cortex	0.474	0.029764	0.037265
	RH precuneus cortex	0.439	0.046413	0.068079
	Left cerebellum cortex	−0.5	0.021014	0.050482
	Right cerebellum cortex	−0.567	0.007292	0.001009

RH, right hemisphere; LH, left hemisphere; DE, designs; VR, visual reproduction.

**FIGURE 4:**
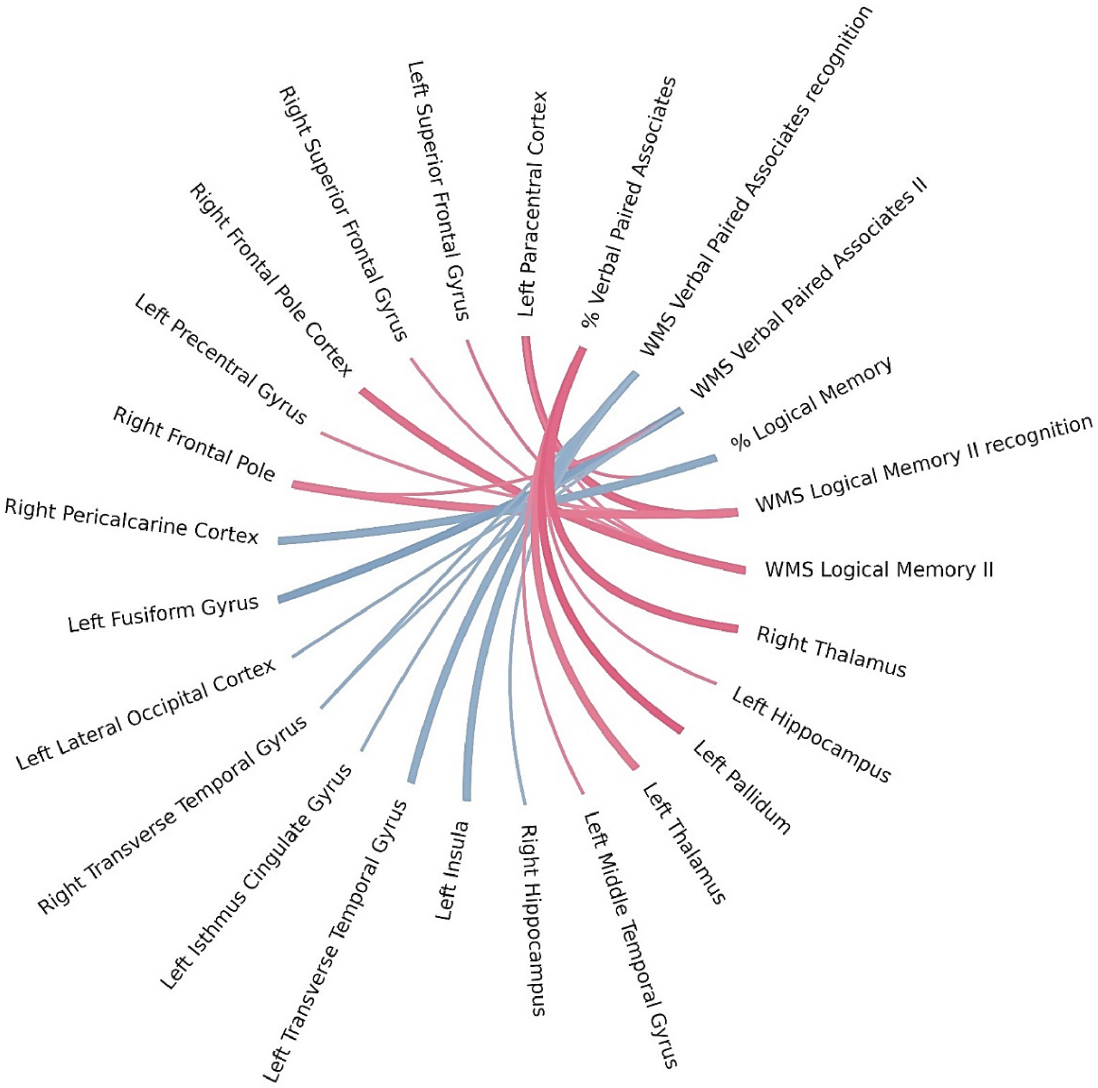
Brain regions correlated with WMS-IV long-term verbal memory indicators in the left-MTLE patient group. The blue lines represent positive correlations and the red lines represent negative correlations, the thickness of the lines reflects the strength of the correlation.

Using Bayesian local FDR correction (threshold < 0.2), we identified significant correlations between long-term verbal memory performance and cortical or subcortical brain structures. Detailed results are presented in Table 5 and Figure 4. Long-term verbal memory indicators were associated with the following regions: fusiform gyrus, isthmus cingulate gyrus, lateral occipital cortex, transverse temporal gyrus, insula, paracentral cortex, precentral gyrus, superior frontal gyrus, middle temporal gyrus, thalamus, pallidum, and hippocampus in the left hemisphere, and the transverse temporal gyrus, pericalcarine cortex, superior frontal gyrus, frontal pole, thalamus, and hippocampus in the right hemisphere.

**TABLE 5:** Gray matter areas associated with long-term verbal memory.

Subtest	Brain region	*R*	*p*	Local FDR
WMS LM II	LH paracentral cortex	−0.449	0.041342	0.177075
	LH superior frontal gyrus	−0.481	0.02729	0.117357
	RH superior frontal gyrus	−0.444	0.043907	0.188958
	RH frontal pole cortex	−0.545	0.010537	0.087175
WMS LM II recognition	LH paracentral cortex	−0.559	0.008389	0.08935
	LH precentral gyrus	−0.448	0.041608	0.178303
	RH frontal pole	−0.51	0.018081	0.091004
% LM	LH paracentral cortex	−0.49	0.024158	0.106654
	RH pericalcarine cortex	0.545	0.010703	0.087016
WMS VPA II	LH fusiform gyrus	0.599	0.004124	0.048965
	LH lateral occipital cortex	0.5	0.021033	0.097607
	RH frontal pole	−0.487	0.025024	0.109467
	RH transverse temporal gyrus	0.476	0.029093	0.124114
WMS VPA II recognition	LH isthmus cingulate gyrus	0.487	0.025269	0.110284
	LH transverse temporal gyrus	0.523	0.014996	0.086778
	LH insula	0.524	0.014812	0.086631
	RH transverse temporal gyrus	0.499	0.021349	0.098435
	Right hippocampus	0.466	0.033208	0.140799
% VPA	LH middle temporal gyrus	−0.455	0.038139	0.16244
	Left thalamus	−0.503	0.020128	0.095358
	Left Pallidum	−0.608	0.003446	0.027508
	Left hippocampus	−0.474	0.029838	0.127014
	Right thalamus	−0.573	0.006567	0.087041

RH, right hemisphere; LH, left hemisphere; LM, logical memory; VPA, verbal paired associates.

**FIGURE 5:**
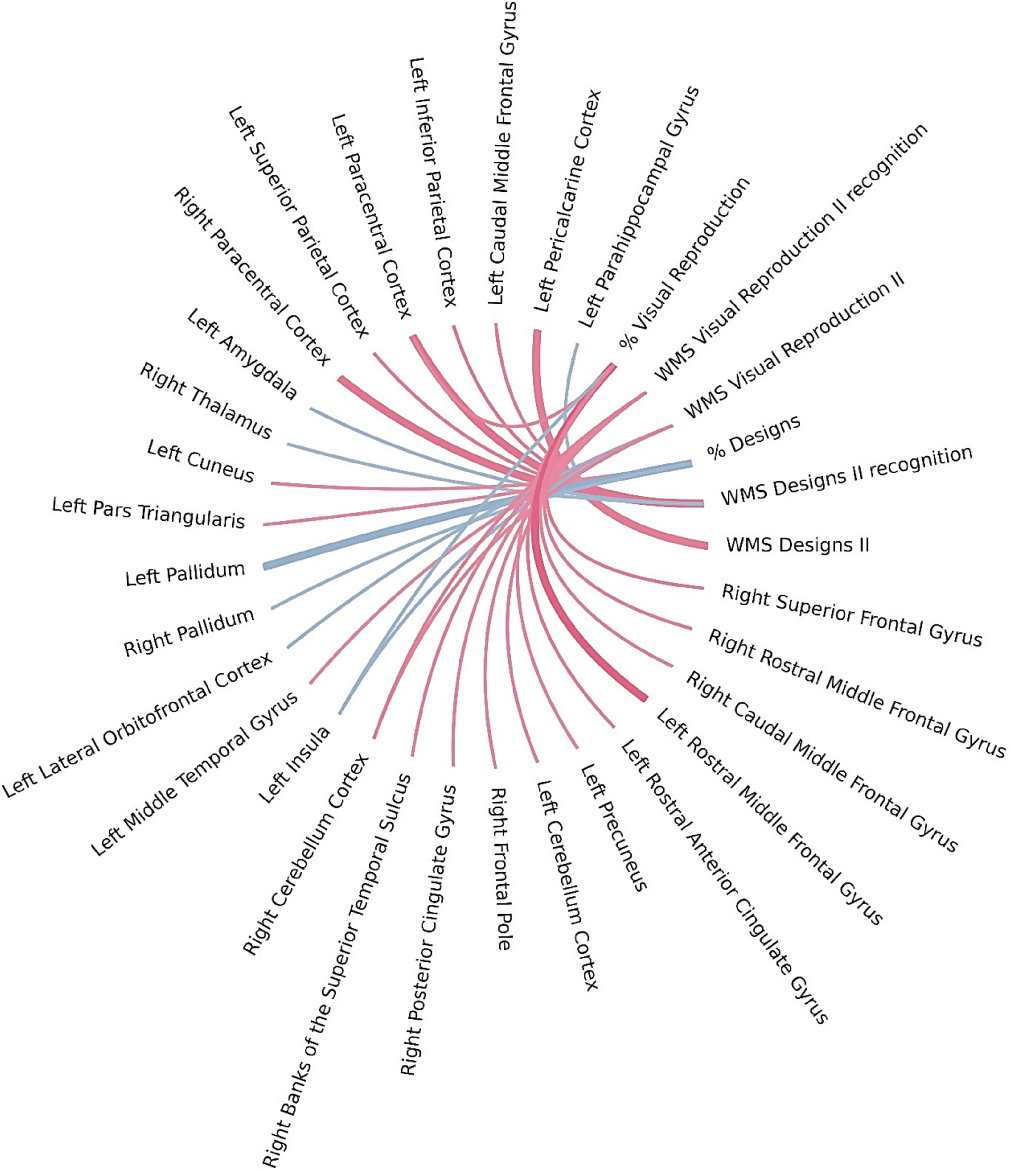
Brain regions correlated with WMS-IV long-term visual memory indicators in the left-MTLE patient group. The blue lines represent positive correlations and the red lines represent negative correlations, the thickness of the lines reflects the strength of the correlation.

As shown in Table 6 and Figure 5, the application of Bayesian local FDR correction (threshold < 0.2) revealed several significant associations between long-term visual memory performance and structural brain measures. The following regions were correlated with long-term visual memory indicators: caudal middle frontal gyrus, lateral orbitofrontal cortex, middle temporal gyrus, precuneus, rostral anterior cingulate gyrus, rostral middle frontal gyrus, inferior parietal cortex, superior parietal cortex, parahippocampal gyrus, paracentral cortex, pars triangularis, pericalcarine cortex, superior frontal gyrus, insula, cuneus, pallidum, and amygdala in the left hemisphere, and caudal middle frontal gyrus, rostral middle frontal gyrus, posterior cingulate gyrus, frontal pole, superior frontal gyrus, paracentral cortex, thalamus, and pallidum in the right hemisphere, along with the cerebellar cortex bilaterally.

**TABLE 6:** Gray matter areas associated with long-term visual memory.

Subtest	Brain region	*r*	*p*	Local FDR
WMS DE II	LH parahippocampal gyrus	0.456	0.037749	0.113731
	LH pericalcarine cortex	−0.507	0.019022	0.121909
WMS DE II recognition	LH caudal middle frontal gyrus	−0.475	0.034313	0.105051
	LH inferior parietal cortex	−0.491	0.02788	0.096946
	LH paracentral cortex	−0.528	0.016818	0.145071
	LH superior parietal cortex	−0.478	0.032931	0.10231
	RH paracentral cortex	−0.524	0.017816	0.133018
	Left Amygdala	0.459	0.041583	0.126123
	Right Thalamus	0.445	0.049561	0.159349
% DE	LH cuneus	−0.447	0.04243	0.129203
	LH pars triangularis	−0.444	0.043726	0.134135
	Left pallidum	0.508	0.018715	0.124431
	Right pallidum	0.453	0.039092	0.11777
WMS VR II	LH lateral orbitofrontal cortex	0.471	0.031157	0.09952
	LH middle temporal gyrus	−0.444	0.043747	0.134217
	LH insula	0.475	0.029506	0.097759
	Right cerebellum cortex	−0.467	0.032872	0.102204
WMS VR II recognition	RH banks STS	−0.467	0.032604	0.101732
	RH posterior cingulate gyrus	−0.452	0.039741	0.119842
	RH frontal pole	−0.456	0.037782	0.113826
	Left cerebellum cortex	−0.461	0.035358	0.107422
	Right cerebellum cortex	−0.48	0.027771	0.096928
% VR	LH paracentral cortex	−0.462	0.034783	0.106087
	LH precuneus	−0.452	0.039663	0.119589
	LH rostral anterior cingulate gyrus	−0.48	0.027756	0.096926
	LH rostral middle frontal gyrus	−0.602	0.003886	0.031644
	LH insula	0.466	0.033169	0.102749
	RH caudal middle frontal gyrus	−0.464	0.033966	0.104319
	RH rostral middle frontal gyrus	−0.468	0.032559	0.101654
	RH superior frontal gyrus	−0.48	0.027495	0.096906

RH, right hemisphere; LH, left hemisphere; DE, designs; VR, visual reproduction.

**FIGURE 6:**
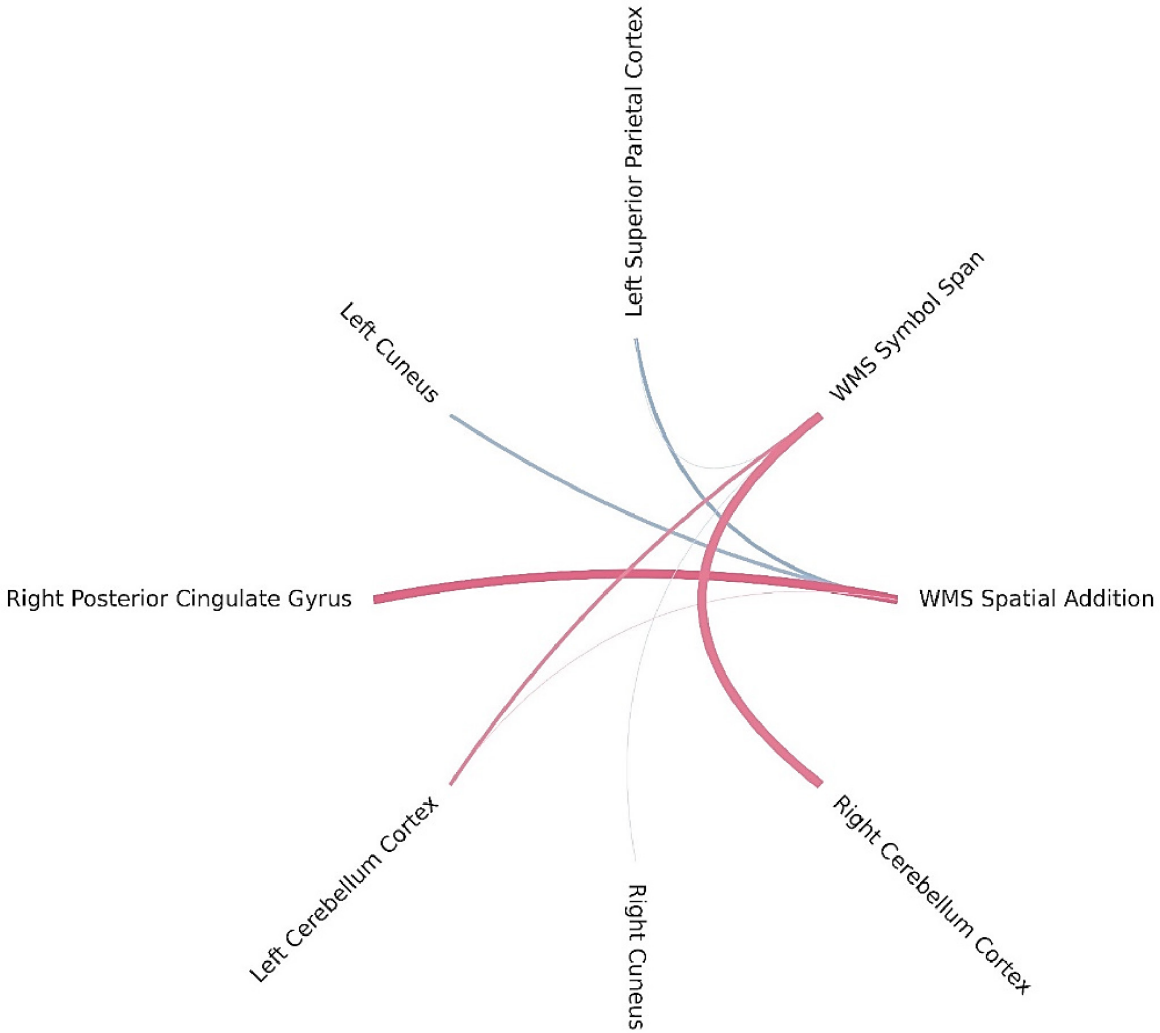
Brain regions correlated with WMS-IV working memory indicators in the left-MTLE patient group. The blue lines represent positive correlations and the red lines represent negative correlations, the thickness of the lines reflects the strength of the correlation.

Bayesian local FDR correction (threshold < 0.2) identified significant correlations between working memory performance and structural brain features (Table 7). The following regions were associated with working memory indicators: the superior parietal cortex in the left hemisphere, the cuneus and posterior cingulate gyrus in the right hemisphere, and the cerebellar cortex bilaterally (Figure 6).

**TABLE 7:** Gray matter areas associated with working memory.

Subtest	Brain region	*r*	*p*	Local FDR
WMS SA	LH superior parietal cortex	0.493	0.023092	0.035573
	LH cuneus	0.442	0.04492	0.112121
	RH posterior cingulate cortex	−0.583	0.005516	0.001799
	Left cerebellum cortex	−0.0668	0.773722	0.006785
WMS SSP	LH superior parietal cortex	0.0778	0.737522	0.140889
	RH cuneus	0.0775	0.738595	0.131409
	Left cerebellum cortex	−0.455	0.038251	0.056754
	Right cerebellum cortex	−0.509	0.018502	0.052504

RH, right hemisphere; LH, left hemisphere; SA, spatial addition; SSP, symbol span.

### 3.5 Association between gray matter volume, memory performance, and clinical variables in left-MTLE population

No significant correlations were found between age at epilepsy onset and any memory indicators. However, epilepsy duration was significantly correlated with two long-term visual memory measures: VR II (*r* = −0.539, *p* = 0.012) and% VR (*r* = −0.491, *p* = 0.024).

By contrast, age at epilepsy onset showed significant negative correlations with gray matter volume in several regions: left inferior temporal cortex (*r* = −0.514, *p* = 0.017), left isthmus cingulate gyrus (*r* = −0.446, *p* = 0.043), left middle temporal gyrus (*r* = −0.464, *p* = 0.034), left thalamus (*r* = −0.434, *p* = 0.049), left pallidum (*r* = −0.497, *p* = 0.022), left hippocampus (*r* = −0.518, *p* = 0.016), right superior frontal gyrus (*r* = −0.449, *p* = 0.041), and right pallidum (*r* = −0.469, *p* = 0.032).

These findings suggest that a longer duration of epilepsy is associated with more severe long-term visual memory deficits, while an older age at epilepsy onset is linked to greater gray matter volume reduction in the specified regions.

## 4 Discussion

This study combined morphometric MRI techniques with neuropsychological testing to assess changes in the functional and structural organization associated with MTLE. The findings clearly show that patients with MTLE exhibit significantly reduced memory functioning, consistent with previous studies on this population (Rayner et al., 2016; Zhu et al., 2021; Novak et al., 2022).

In analyzing gray matter abnormalities, we found that patients with left-MTLE showed significant alterations in the volumes of specific brain regions. These changes were observed not only near the epileptic focus but also in more distant brain areas. Notably, volume reductions were found in the temporal lobe (superior temporal gyrus and temporal pole) and in more remote regions, such as the right frontal lobe (pars orbitalis) and the right globus pallidus. A decrease in gray matter volume was also observed bilaterally in the cerebellar cortex—findings consistent with previous research (Colnaghi et al., 2017; Zhang et al., 2017). Although other studies have reported bilateral thalamic volume reductions in patients with MTLE (Wei et al., 2016; Zheng et al., 2018), this was not observed in the present study.

In addition, compared with healthy participants, patients with left-MTLE showed increased volume in the cuneus and the lateral part of the occipital lobe in the left hemisphere (the side of the epileptic focus), as well as in the caudal anterior cingulate cortex, posterior cingulate cortex, caudate nucleus, and amygdala in the right hemisphere (contralateral to the epileptic focus). As demonstrated in the study by Zubal et al. (2025), this hypertrophy—particularly in the contralateral amygdala—may result from the negative impact of seizures and the brain’s compensatory response to cognitive deficits. Other studies have also reported amygdala enlargement in temporal lobe epilepsy. While some of these focused on the amygdala ipsilateral to the epileptic focus (Kim et al., 2012; Mitsueda-Ono et al., 2011; Bower et al., 2003), three studies have documented enlargement in the contralateral amygdala (Coan et al., 2013; Whelan et al., 2018; Zubal et al., 2025). However, only one of these (Zubal et al., 2025) linked this finding to memory impairment.

Although we did not find a correlation between contralateral amygdala volume and memory performance, as reported by Zubal et al. (2025), it is possible that the hypertrophy observed in other regions—both ipsilateral and contralateral to the epileptic focus—may have arisen through a similar compensatory mechanism. In light of this, the detection of negative correlations between memory function and the volume of certain brain regions is particularly noteworthy.

For the WMS LM I task, significant negative correlations were observed with the left middle temporal gyrus, superior frontal gyrus, pars orbitalis, and right frontal pole, suggesting that reduced cortical thickness in these regions may be associated with better immediate verbal memory recall. Interestingly, a positive correlation was also found with the left insula, indicating potential region-specific contributions to encoding or attentional control. The WMS VPA I task showed positive correlations with the left fusiform gyrus, left insula, and right transverse temporal gyrus—regions well known for their involvement in auditory-verbal processing (Liegeois-Chauvel et al., 1991; Skipper and Small, 2006; Price, 2010). By contrast, negative correlations were observed with the right frontal pole and parahippocampal cortex. These findings are not entirely consistent with other studies, which report that the ability to temporarily maintain speech-related information is associated with the left insula as well as other regions in the dominant hemisphere, such as the inferior frontal premotor regions, parietal cortex, and Broca’s area (Vallar, 2006; Cascella and Al Khalili, 2024)—components of the language system that are clearly linked to the verbal modality of the task (Liegeois-Chauvel et al., 1991; Skipper and Small, 2006; Price, 2010). Additionally, research by Ćurčić-Blake et al. (2017) highlights that inhibitory deficits could contribute to failures in controlling the contents of memory and that the putamen is involved in translating memories into language experience—findings consistent with our results, which suggest a complex interplay between fronto-limbic and temporal regions during associative verbal learning. For short-term visual memory, positive correlations were observed with the posterior cingulate gyrus, rostral anterior cingulate gyrus, fusiform gyrus, precuneus, and superior parietal cortex, underscoring the role of visuospatial integration, object recognition, and attention control in short-term visual reproduction. Additionally, negative correlations were found between WMS DE I and WMS VR I scores and the volume of the left and right cerebellar cortex, which may reflect reduced reliance on subcortical motor planning during efficient visual encoding. Our findings are consistent with studies on the neuronal correlates of visual short-term memory, which highlight the involvement of the occipital extrastriate cortex, posterior parietal cortex, prefrontal cortex, dorsal frontal area, parietal lobule, frontal premotor regions, and insula (Zimmer, 2008; Cascella and Al Khalili, 2024). Interestingly, our study found correlations between short-term memory and the insula only in the verbal modality. The association with the cerebellar cortex may be explained by the simultaneous involvement of working memory, which could be due to the complexity of the neuropsychological tasks.

The structure most commonly emphasized in long-term declarative memory is the hippocampus, where the consolidation of information from short-term to long-term memory takes place (Almaraz-Espinoza and Grider, 2023). The amygdala, by contrast, is associated with the emotional aspects of remembered information, which are further stored throughout the cortex (Almaraz-Espinoza and Grider, 2023). Some studies also highlight the role of prefrontal regions in memory recall (Braver et al., 2001). The results obtained in the present research are consistent with these findings, particularly in the domain of visual long-term memory: significant correlations were observed with prefrontal executive regions, temporo-parietal regions, the insula, pallidum, and amygdala in the left hemisphere, as well as the thalamus and pallidum in the right hemisphere.

It should be noted that we found positive correlations primarily in the hemisphere ipsilateral to the epileptic focus. In terms of long-term verbal memory, the results included both positive and negative correlations across a range of cortical and subcortical regions. Notably, lower scores on the% VPA task were associated with reduced volumes in the left and right thalamus, left pallidum, and left hippocampus, suggesting a potential role for subcortical memory circuits in verbal learning (O’Mara and Aggleton, 2019). Positive associations were observed between WMS VPA II recognition and cortical thickness in regions such as the insula, transverse temporal gyrus, and cingulate isthmus, while WMS VPA II showed both positive and negative correlations with occipital, temporal, and notably frontal areas—structures more commonly linked to short-term memory (Nee et al., 2013; Eriksson et al., 2015). Performance on WMS LM II and WMS LM II recognition was negatively correlated with thickness in the frontal pole, superior frontal gyrus, paracentral, and precentral regions, suggesting inefficiency of frontal-executive structures in narrative memory retrieval (Meléndez et al., 2019). Additionally, % LM scores were associated with cortical thickness in both the occipital and paracentral cortices, emphasizing the role of fronto-temporo-limbic networks in long-term verbal memory. A particularly intriguing finding was the negative correlation between long-term verbal memory and the left hippocampus, alongside a positive correlation with the right hippocampus in patients with left-MTLE. This may indicate that as the left hippocampus becomes smaller and less functional, the right hippocampus, through neuronal reorganization and plasticity, increasingly assumes its functions. As a result, enlargement of the right hippocampus in left-MTLE may reflect compensatory adaptation, and greater right hippocampal volume may correspond with better memory performance because it takes on the role of supporting verbal memory. A study by Chen et al. (2017), which used resting-state functional connectivity (RSFC), supports this interpretation. Their findings showed that in individuals with left-MTLE, enhanced interhemispheric RSFC between the left hippocampus/amygdala and the right hippocampus was associated with better verbal memory, while increased RSFC between the left hippocampus and the left postcentral gyrus was linked to poorer verbal memory performance.

It should also be noted that some studies suggest a thinner cerebral cortex may indicate more effective and efficient neuronal systems (Menary et al., 2013; Wagstyl and Lerch, 2018). Thus, the results obtained in our study—showing a thinner cortex in patients with left-MTLE—can be interpreted in two distinct ways. A longitudinal study in patients with MTLE, collecting data on changes in various aspects of memory at different stages of the disease and taking into account neuroanatomical alterations, could help clarify which interpretation is more accurate.

For WMS SA, higher scores were positively associated with the left superior parietal and right cuneus cortical thickness and negatively with the right posterior cingulate cortex. These regions are classically involved in visuospatial integration and attentional regulation (Leech and Sharp, 2014), suggesting that efficient activation of parietal and cingulate regions supports spatial memory processing. The WMS SSP task showed similar involvement of parietal and occipital regions, with significant positive correlations in the left superior parietal and right cuneus and negative correlations with the left and right cerebellar cortex, suggesting a potential modulatory role of cerebellar–cortical networks in spatial working memory tasks. The left hemisphere superior parietal area and right hemisphere cuneus correlated positively with working memory, which is consistent with the study by Eriksson et al. (2015), who indicate that the superior parietal cortex is involved in the executive aspects of working memory and with working memory capacity. However, they indicated a strong connection not of the left, but of the right parietal cortex with the spatial working memory task, which is the one used in this study (Eriksson et al., 2015). Furthermore, we did not find a correlation with the prefrontal cortex, which is highlighted as critical in working memory, especially the dorsal PFC, as the one more strongly involved in spatial working memory task (Ptak, 2012; Nee et al., 2013; Eriksson et al., 2015). Although we did not find a correlation with the prefrontal cortex, our findings—along with those of other studies—support the idea that a network of frontal and parietal cortical areas, along with the cerebellum (Brissenden and Somers, 2019), collectively referred to as the dorsal attention network, supports visual attentional function (Ptak, 2012).

An interesting study was conducted by Zhang et al. (2017), who described the hippocampus-associated causal network of structural damage in temporal lobe epilepsy. In this study, the researchers examined gray matter changes in patients with MTLE, depending on the duration of epilepsy and the estimated number of lifetime seizures. They introduced the term Granger Causality (GC), which measures the strength of effective connectivity—representing causal interactions by assessing how activity in one neural region influences another. It quantifies the extent to which the signal from a seed region can predict the signal in a target region (Vanneste et al., 2021; Granger, 1969). The authors observed that the lateral temporal lobe ipsilateral to the epileptogenic focus, bilateral lateral prefrontal cortex, medial prefrontal cortex, insula, and thalamus showed consistently positive GC values, while the contralateral temporal cortex, posterior cingulate cortex, and amygdala showed consistently negative GC values. The bilateral basal ganglia, including the caudate head and putamen, were characterized by divergent GC values across different analyses.

A positive GC value indicates that the reduction in gray matter volume within a region is causally linked to, and follows, hippocampal atrophy—suggesting a potential seizure-related damaging effect originating from the hippocampus. By contrast, a negative GC value indicates that a region shows an opposing (increased) gray matter volume alteration in response to hippocampal atrophy, which is interpreted as a compensatory structural effect (Zhang et al., 2017). In our study, we observed a decrease in gray matter volume in the lateral temporal lobe ipsilateral to the epileptogenic focus, the contralateral prefrontal cortex, and the ipsilateral hippocampus—findings that likely reflect damage driven by hippocampal atrophy. Presumably, this degeneration contributes to increased cognitive deficits. Conversely, an observed increase in the contralateral posterior cingulate cortex and amygdala may represent a protective or compensatory response. This supports the idea of a complex repair mechanism in which less affected brain regions are recruited to support cognitive functioning. Further evidence of this process comes from the positive correlations we observed between memory indicators and the right hemisphere transverse temporal gyrus and pallidum, which may suggest that functional reorganization in these areas has already occurred. On the other hand, the negative correlation between memory indicators and the contralateral posterior cingulate cortex—despite its increased volume in patients with epilepsy—may indicate that while structural reorganization has taken place, functional reorganization in this region has yet to follow.

These findings emphasize the involvement of fronto-temporo-limbic networks in memory and support the utility of local FDR methods in uncovering subtle yet meaningful brain–behavior relationships that may be overlooked using more conservative correction procedures. Although none of these results survived classical FDR correction, the application of local FDR revealed potentially meaningful effects that would otherwise remain undetected. The findings underscore the role of frontal, temporal, and limbic structures in supporting different types of memory performance. Additionally, several subcortical regions—such as the thalamus, pallidum, and hippocampus—also emerged, suggesting the involvement of broader memory-related networks.

Although some studies have evaluated memory (Butler and Zeman, 2008; Baulac, 2015; Rayner et al., 2016; Zhu et al., 2021) and brain abnormalities in patients with MTLE (Bonilha et al., 2012; Xie et al., 2024; Ishizaki et al., 2025), only a few have combined the two approaches (Cabalo et al., 2024; Park et al., 2017). Furthermore, there is a scarcity of studies that collect such detailed data on the various facets of memory in patients with epilepsy, which allow for a comprehensive examination of patients’ memory profiles and their relationship to anatomical correlates. Our study attempts to address this gap.

## 5 Conclusion

This work enriches our understanding of the specificity of the memory system in patients with temporal lobe epilepsy. Patients performed worse than the control group, exhibiting a broad range of deficits across all assessed types and modalities of memory. Particularly noteworthy is the study’s identification of a distinct pattern of neuronal aberrations and correlations between specific brain structures and memory performance. The use of Bayesian local FDR correction enabled the detection of meaningful structure–function relationships that may have gone unnoticed with more conservative correction methods. These findings suggest that the degree of structural brain anomalies may be linked to the extent of memory impairment in temporal lobe epilepsy, highlighting the complex interplay between neuroanatomy and cognitive function. Some results from this study support a tentative model: an initial structural reduction leads to cognitive difficulty, followed by structural reorganization, and eventually functional reorganization that may enable cognitive improvement. Therefore, even when increases in certain brain structures are observed—previously reported in other studies as potentially protective—this does not necessarily translate into improved cognitive outcomes. Further research is needed to clarify the mechanisms underlying these memory deficits. Given the limited sample size in the present study, replication and extension through longitudinal research would be valuable for observing structural and mnestic changes over time. Such studies could help determine whether hypertrophy in specific structures results from bioelectrical abnormalities associated with seizures or reflects a plasticity-driven reorganization of brain networks aimed at mitigating memory or broader cognitive deficits.

## Data Availability

The raw data supporting the conclusions of this article will be made available by the authors, without undue reservation.

## References

[B1] Almaraz-Espinoza A. Grider M. H. (2023). “Physiology, long term memory,” in *StatPearls.* Treasure Island, FL: StatPearls Publishing. 31747198

[B2] Bala A. Olejnik A. Mojżeszek M. Rysz A. Kunert P. (2024). Navigating social waters: Understanding theory-of-mind challenges in patients with mesial temporal lobe epilepsy. *J. Clin. Med.* 13:1410. 10.3390/jcm13051410 38592233 PMC10932399

[B3] Baulac M. (2015). MTLE with hippocampal sclerosis in adult as a syndrome. *Revue Neurol.* 171 259–266. 10.1016/j.neurol.2015.02.004 25727907

[B4] Bell B. D. Seidenberg M. Hermann B. P. Douville K. (2003). Visual and auditory naming in patients with left or bilateral temporal lobe epilepsy. *Epilepsy Res.* 55 29–37. 10.1016/s0920-1211(03)00110-4 12948614

[B5] Bonilha L. Nesland T. Martz G. U. Joseph J. E. Spampinato M. V. Edwards J. C. (2012). Medial temporal lobe epilepsy is associated with neuronal fibre loss and paradoxical increase in structural connectivity of limbic structures. *J. Neurol. Neurosurg. Psychiatry* 83 903–909. 10.1136/jnnp-2012-302476 22764263 PMC3415309

[B6] Bower S. P. Vogrin S. J. Morris K. Cox I. Murphy M. Kilpatrick C. J. (2003). Amygdala volumetry in “imaging-negative” temporal lobe epilepsy. *J. Neurol. Neurosurg. Psychiatry* 74 1245–1249. 10.1136/jnnp.74.9.1245 12933928 PMC1738652

[B7] Braver T. S. Barch D. M. Kelley W. M. Buckner R. L. Cohen N. J. Miezin F. M. (2001). Direct comparison of prefrontal cortex regions engaged by working and long-term memory tasks. *NeuroImage* 14 48–59. 10.1006/nimg.2001.0791 11525336

[B8] Brissenden J. A. Somers D. C. (2019). Cortico-cerebellar networks for visual attention and working memory. *Curr. Opin. Psychol.* 29 239–247. 10.1016/j.copsyc.2019.05.003 31202085 PMC7256875

[B9] Butler C. R. Zeman A. Z. (2008). Recent insights into the impairment of memory in epilepsy: Transient epileptic amnesia, accelerated long-term forgetting and remote memory impairment. *Brain J. Neurol.* 131 2243–2263. 10.1093/brain/awn127 18669495

[B10] Cabalo D. G. DeKraker J. Royer J. Xie K. Tavakol S. Rodríguez-Cruces R. (2024). Differential reorganization of episodic and semantic memory systems in epilepsy-related mesiotemporal pathology. *Brain J. Neurol.* 147 3918–3932. 10.1093/brain/awae197 39054915 PMC11531848

[B11] Caciagli L. Paquola C. He X. Vollmar C. Centeno M. Wandschneider B. (2023). Disorganization of language and working memory systems in frontal versus temporal lobe epilepsy. *Brain J. Neurol.* 146 935–953. 10.1093/brain/awac150 35511160 PMC9976988

[B12] Cascella M. Al Khalili Y. (2024). “Short-term memory impairment,” in *StatPearls.* Treasure Island, FL: StatPearls Publishing. 31424720

[B13] Chen S. Adepu M. Emady H. Jiao Y. Gel A. (2017). Enhancing the physical modeling capability of open-source MFIX-DEM software for handling particle size polydispersity: Implementation and validation. *Powder Technol.* 317:55. 10.1016/j.powtec.2017.04.055

[B14] Coan A. C. Morita M. E. Campos B. M. Bergo F. P. Kubota B. Y. Cendes F. (2013). Amygdala enlargement occurs in patients with mesial temporal lobe epilepsy and hippocampal sclerosis with early epilepsy onset. *Epilepsy Behav.* 29 390–394. 10.1016/j.yebeh.2013.08.022 24074891

[B15] Colnaghi S. Beltrami G. Poloni G. Pichiecchio A. Bastianello S. Galimberti C. A. (2017). Parahippocampal involvement in mesial temporal lobe epilepsy with hippocampal sclerosis: A proof of concept from memory-guided saccades. *Front. Neurol.* 8:595. 10.3389/fneur.2017.00595 29163352 PMC5681931

[B16] Ćurčić-Blake B. Ford J. M. Hubl D. Orlov N. D. Sommer I. E. Waters F. (2017). Interaction of language, auditory and memory brain networks in auditory verbal hallucinations. *Progr. Neurobiol.* 148 1–20. 10.1016/j.pneurobio.2016.11.002 27890810 PMC5240789

[B17] Dale A. M. Sereno M. I. (1993). Improved localizadon of cortical activity by combining EEG and MEG with MRI cortical surface reconstruction: A linear approach. *J. Cogn. Neurosci.* 5 162–176. 10.1162/jocn.1993.5.2.162 23972151

[B18] Dale A. M. Fischl B. Sereno M. I. (1999). Cortical surface-based analysis. I. Segmentation and surface reconstruction. *NeuroImage* 9 179–194. 10.1006/nimg.1998.0395 9931268

[B19] Desikan R. S. Ségonne F. Fischl B. Quinn B. T. Dickerson B. C. Blacker D. (2006). An automated labeling system for subdividing the human cerebral cortex on MRI scans into gyral based regions of interest. *NeuroImage* 31 968–980. 10.1016/j.neuroimage.2006.01.021 16530430

[B20] Dos Santos, Silva R. P. Lima Angelo I. C. B. De Medeiros Dantas G. C. De Souza J. M. Pinheiro Pessoa J. R. C. (2024). Pattern of abnormalities on gray matter in patients with medial temporal lobe epilepsy and hippocampal sclerosis: An updated meta-analysis. *Clin. Neurol. Neurosurg.* 245:108473. 10.1016/j.clineuro.2024.108473 39154538

[B21] Eriksson J. Vogel E. K. Lansner A. Bergström F. Nyberg L. (2015). Neurocognitive architecture of working memory. *Neuron* 88 33–46. 10.1016/j.neuron.2015.09.020 26447571 PMC4605545

[B22] Fischl B. Dale A. M. (2000). Measuring the thickness of the human cerebral cortex from magnetic resonance images. *Proc. Natl. Acad. Sci. U S A.* 97 11050–11055. 10.1073/pnas.200033797 10984517 PMC27146

[B23] Fischl B. Salat D. H. Busa E. Albert M. Dieterich M. Haselgrove C. (2002). Whole brain segmentation: Automated labeling of neuroanatomical structures in the human brain. *Neuron* 33 341–355. 10.1016/s0896-6273(02)00569-x 11832223

[B24] Fischl B. Sereno M. I. Dale A. M. (1999). Cortical surface-based analysis. II: Inflation, flattening, and a surface-based coordinate system. *NeuroImage* 9 195–207. 10.1006/nimg.1998.0396 9931269

[B25] Granger C. W. J. (1969). Investigating causal relations by econometric models and cross-spectral methods. *Econometrica* 37 424–438. 10.2307/1912791

[B26] Ishizaki T. Maesawa S. Suzuki T. Hashida M. Ito Y. Yamamoto H. (2025). Frequency-specific network changes in mesial temporal lobe epilepsy: Analysis of chronic and transient dysfunctions in the temporo-amygdala-orbitofrontal network using magnetoencephalography. *Epilepsia Open* 10 557–570. 10.1002/epi4.70018 40047314 PMC12014939

[B27] Ives-Deliperi V. Butler J. T. (2021). Mechanisms of cognitive impairment in temporal lobe epilepsy: A systematic review of resting-state functional connectivity studies. *Epilepsy Behav.* 115:107686. 10.1016/j.yebeh.2020.107686 33360743

[B28] Kim D. W. Lee S. K. Chung C. K. Koh Y. C. Choe G. Lim S. D. (2012). Clinical features and pathological characteristics of amygdala enlargement in mesial temporal lobe epilepsy. *J. Clin. Neurosci.* 19 509–512. 10.1016/j.jocn.2011.05.042 22321366

[B29] Kwan P. Sander J. W. (2004). The natural history of epilepsy: An epidemiological view. *J. Neurol. Neurosurg. Psychiatry* 75 1376–1381. 10.1136/jnnp.2004.045690 15377680 PMC1738749

[B30] Leech R. Sharp D. J. (2014). The role of the posterior cingulate cortex in cognition and disease. *Brain J. Neurol.* 137 12–32. 10.1093/brain/awt162 23869106 PMC3891440

[B31] Li X. Morgan P. S. Ashburner J. Smith J. Rorden C. (2016). The first step for neuroimaging data analysis: DICOM to NIfTI conversion. *J. Neurosci. Methods* 264 47–56. 10.1016/j.jneumeth.2016.03.001 26945974

[B32] Liegeois-Chauvel C. Musolino A. Chauvel P. (1991). Localization of the primary auditory area in man. *Brain* 114 139–151. 10.1093/oxfordjournals.brain.a101854 1900211

[B33] Meléndez J. C. Redondo R. Escudero J. Satorres E. Pitarque A. (2019). Executive functions, episodic autobiographical memory, problem-solving capacity, and depression proposal for a structural equations model. *J. Geriatr. Psychiatry Neurol.* 32 81–89. 10.1177/0891988718824037 30665320

[B34] Menary K. Collins P. F. Porter J. N. Muetzel R. Olson E. A. Kumar V. (2013). Associations between cortical thickness and general intelligence in children, adolescents and young adults. *Intelligence* 41 597–606. 10.1016/j.intell.2013.07.010 24744452 PMC3985090

[B35] Mitsueda-Ono T. Ikeda A. Inouchi M. Takaya S. Matsumoto R. Hanakawa T. (2011). Amygdalar enlargement in patients with temporal lobe epilepsy. *J. Neurol. Neurosurg. Psychiatry* 82 652–657. 10.1136/jnnp.2010.206342 21047879

[B36] Nee D. E. Brown J. W. Askren M. K. Berman M. G. Demiralp E. Krawitz A. (2013). A meta-analysis of executive components of working memory. *Cereb. Cortex* 23 264–282. 10.1093/cercor/bhs007 22314046 PMC3584956

[B37] Novak A. Vizjak K. Rakusa M. (2022). Cognitive impairment in people with epilepsy. *J. Clin. Med.* 11:267. 10.3390/jcm11010267 35012007 PMC8746065

[B38] Olejnik A. Bala A. Dziedzic T. Rysz A. Marchel A. Kunert P. (2024). Executive dysfunction profile in mesial temporal lobe epilepsy. *Acta Neuropsychol.* 22 1–13. 10.5604/01.3001.0053.9737

[B39] O’Mara S. M. Aggleton J. P. (2019). Space and memory (Far) beyond the hippocampus: Many subcortical structures also support cognitive mapping and mnemonic processing. *Front. Neural Circuits* 13:52. 10.3389/fncir.2019.00052 31447653 PMC6692652

[B40] Ono S. E. Mader-Joaquim M. J. de Carvalho Neto A. de Paola L. Dos Santos G. R. Silvado C. E. S. (2021). Relationship between hippocampal subfields and verbal and visual memory function in mesial temporal lobe epilepsy patients. *Epilepsy Res.* 175:106700. 10.1016/j.eplepsyres.2021.106700 34175793

[B41] Pail M. Brázdil M. Marecek R. Mikl M. (2010). An optimized voxel-based morphometric study of gray matter changes in patients with left-sided and right-sided mesial temporal lobe epilepsy and hippocampal sclerosis (MTLE/HS). *Epilepsia* 51, 511–518. 10.1111/j.1528-1167.2009.02324.x 19817822

[B42] Park C. H. Choi Y. S. Jung A. R. Chung H. K. Kim H. J. Yoo J. H. (2017). Seizure control and memory impairment are related to disrupted brain functional integration in temporal lobe epilepsy. *J. Neuropsychiatry Clin. Neurosci.* 29 343–350. 10.1176/appi.neuropsych.16100216 28449635

[B43] Price C. J. (2010). The anatomy of language: A review of 100 fMRI studies published in 2009. *Ann. N. Y. Acad. Sci.* 1191 62–88. 10.1111/j.1749-6632.2010.05444.x 20392276

[B44] Ptak R. (2012). The frontoparietal attention network of the human brain: Action, saliency, and a priority map of the environment. *Neurosci. Rev. J. Bringing Neurobiol. Neurol. Psychiatry* 18 502–515. 10.1177/1073858411409051 21636849

[B45] Rayner G. Jackson G. D. Wilson S. J. (2016). Mechanisms of memory impairment in epilepsy depend on age at disease onset. *Neurology* 87 1642–1649. 10.1212/WNL.0000000000003231 27638925 PMC5085077

[B46] Ryvlin P. Cross J. H. Rheims S. (2014). Epilepsy surgery in children and adults. *Lancet Neurol.* 13 1114–1126. 10.1016/S1474-4422(14)70156-5 25316018

[B47] Siemianowski C. Królicki L. (2005). Znaczenie metod neuroobrazowania w diagnostyce padaczek. *Pol. Przegl. Neurol.* 1 76–80.

[B48] Skipper J. I. Small S. L. (2006). “fMRI studies of language,” in *Encyclopedia of Language & Linguistics.* Amsterdam: Elsevier Ltd, 496–511. 10.1016/B0-08-044854-2/02399-3

[B49] Vallar G. (2006). Memory systems: The case of phonological short-term memory. A festschrift for Cognitive Neuropsychology. *Cogn. Neuropsychol.* 23 135–155. 10.1080/02643290542000012 21049325

[B50] Vanneste S. Mohan A. De Ridder D. To W. T. (2021). The BDNF Val^66^Met polymorphism regulates vulnerability to chronic stress and phantom perception. *Prog. Brain Res.* 260 301–326. 10.1016/bs.pbr.2020.08.005 33637225

[B51] Wagstyl K. Lerch J. P. (2018). *Cortical Thickness. Brain Morphometry.* Berlin: Springer, 35–49. 10.1007/978-1-4939-7647-8_3

[B52] Wechsler D. (2009). *Wechsler Memory Scale*, 4th Edn. San Antonio, TX: Pearson.

[B53] Wei W. Zhang Z. Xu Q. Yang F. Sun K. Lu G. (2016). More severe extratemporal damages in mesial temporal lobe epilepsy with hippocampal sclerosis than that with other lesions: A multimodality MRI study. *Medicine* 95:e3020. 10.1097/MD.0000000000003020 26962820 PMC4998901

[B54] Whelan C. D. Altmann A. Botía J. A. Jahanshad N. Hibar D. P. Absil J. (2018). Structural brain abnormalities in the common epilepsies assessed in a worldwide ENIGMA study. *Brain J. Neurol.* 141 391–408. 10.1093/brain/awx341 29365066 PMC5837616

[B55] Xie K. Royer J. Larivière S. Rodriguez-Cruces R. Frässle S. Cabalo D. G. (2024). Atypical connectome topography and signal flow in temporal lobe epilepsy. *Progr. Neurobiol.* 236:102604. 10.1016/j.pneurobio.2024.102604 38604584

[B56] Zhang Z. Liao W. Xu Q. Wei W. Zhou H. J. Sun K. (2017). Hippocampus-associated causal network of structural covariance measuring structural damage progression in temporal lobe epilepsy. *Hum. Brain Mapp.* 38 753–766. 10.1002/hbm.23415 27677885 PMC6866709

[B57] Zheng L. Bin G. Zeng H. Zou D. Gao J. Zhang J. (2018). Meta-analysis of voxel-based morphometry studies of gray matter abnormalities in patients with mesial temporal lobe epilepsy and unilateral hippocampal sclerosis. *Brain Imaging Behav.* 12 1497–1503. 10.1007/s11682-017-9797-5 29302917

[B58] Zhou X. Zhang Z. Liu J. Qin L. Pang X. Zheng J. (2019). Disruption and lateralization of cerebellar-cerebral functional networks in right temporal lobe epilepsy: A resting-state fMRI study. *Epilepsy Behav.* 96 80–86. 10.1016/j.yebeh.2019.03.020 31103016

[B59] Zhu G. Wang J. Xiao L. Yang K. Huang K. Li B. (2021). Memory deficit in patients with temporal lobe epilepsy: Evidence from eye tracking technology. *Front. Neurosci.* 15:716476. 10.3389/fnins.2021.716476 34557066 PMC8453169

[B60] Zimmer H. D. (2008). Visual and spatial working memory: From boxes to networks. *Neurosci. Biobehav. Rev.* 32 1373–1395. 10.1016/j.neubiorev.2008.05.016 18603299

[B61] Zubal R. Velicky Buecheler M. Sone D. Postma T. De Tisi J. Caciagli L. (2025). Brain hypertrophy in patients with mesial temporal lobe epilepsy with hippocampal sclerosis and its clinical correlates. *Neurology* 104:e210182. 10.1212/WNL.0000000000210182 39715478 PMC11666274

